# Association between nutrition literacy and hypertension control: chain mediating association of diet-control intention and salt-restriction tools utilization

**DOI:** 10.3389/fnut.2026.1722719

**Published:** 2026-02-19

**Authors:** Bingyong Zhang, Wending Yin, Rumeng Liu, Huaqing Liu

**Affiliations:** School of Public Health, Bengbu Medical University, Bengbu, China

**Keywords:** blood pressure control, chain mediation, diet-control intention, hypertension, nutritional literacy, salt-restriction tool utilization

## Abstract

**Introduction:**

The limited success in controlling hypertension highlights its role as a persistent and critical public health issue in the Chinese population. Nutritional literacy (NL), as a modifiable factor, may influence blood pressure management through diet-control intention and salt-restriction tool utilization.

**Methods:**

Date came from a cross-sectional survey conducted in Bengbu in 2023. Using multi-stage stratified random sampling, 866 hypertensive adults were enrolled. Data on demographics, NL, diet-control intention, salt-restriction tool use, and self-rated hypertension control were collected via structured questionnaires. Preacher & Hayes four-path framework were applied to examine chain mediation, with significance assessed by bias-corrected bootstrap.

**Results:**

Higher NL was significantly associated with stronger diet-control intention (OR = 1.11, 95% CI: 1.09–1.14), greater salt-restriction tool utilization (OR = 1.08, 95% CI: 1.05–1.12), and better self-rated hypertension control (OR = 1.04, 95% CI: 1.02–1.06). Mediation analysis showed that the association between NL and hypertension control was partly accounted for by salt-restriction tool use (β = −0.0026, 95% CI: −0.0046 to −0.0006) and by a sequential pathway through diet-control intention and salt-restriction tool use (β = −0.0008, 95% CI: −0.0015 to −0.0001).

**Conclusion:**

NL is positively associated with hypertension control, with chain mediating the associations of diet-control intention and salt-restriction tool utilization. Enhancing NL and strengthening dietary self-regulation behaviors may provide associative strategies for hypertension prevention and management.

## 1 Introduction

Globally, hypertension stands out as one of the most widespread chronic non-communicable disorders, and it has long been recognized as a principal driver of cardiovascular morbidity and excess mortality (1). Estimates from 2018 suggested that nearly 1.3 billion adults were living with hypertension worldwide, and the prevalence has shown a steady upward trend (2). In China, hypertension prevalence has risen markedly, reaching 31.6% among adults, with the frequency increasing substantially across older age groups (3). Projections indicate that by 2030 the proportion of elderly individuals with hypertension in China may surpass 50%. Hypertension now constitutes a significant public health concern in China, adversely influencing population health and further intensifying the overall disease burden.

Even with improvements in hypertension prevention and therapy, the overall population-level control outcomes are still far from optimal. In China, only 16.8% of hypertensive patients achieve adequate control (4). There are a multitude of factors influence the effectiveness of hypertension control, such as genetics, behaviors, and environment. Notably, dietary nutrition, a key behavioral factor that can be modified through interventions, plays a pivotal role in blood pressure regulation (5). Extensive evidence indicates that a dietary pattern high in sodium intake, low in potassium and fiber intake, and high in saturated fat intake closely linked with hypertension. Adjustments in dietary patterns have demonstrated effectiveness in reducing blood pressure and decreasing the likelihood of cardiovascular complications.

Excessive salt intake represents a major modifiable determinant of hypertension. In China, although the mean daily salt intake had declined from 10.5 g in 2012 to 9.3 g in 2018 (6), it has remained at a relatively high level. The available evidence indicates that both population-level salt restriction and individual-level salt-control tools have the capacity to translate directly into enhanced blood pressure control (7, 8). However, only 8% of households were using salt-restriction tools. Fortunately, intensive intervention program makes this figure increase to 32%, indicating a significant opportunity for improvement. Conversely, there was a notable rise in awareness of low-sodium salt, from 29 to 45%. However, this increase was not reflected in the actual adoption of low-sodium salt (8).

Nutritional literacy (NL), situated at the intersection of nutrition science and health literacy, regards as an individual's capacity to access, comprehend, and utilize nutrition information in order to make informed dietary choices (9). Over the past decade, NL has received increasing scholarly interest within health promotion research, accompanied by the creation of various instruments designed to assess nutritional literacy (10, 11). Individuals with high nutritional literacy are often capable of interpreting food labels and assessing dietary risks, thereby reducing intake of processed foods, takeaways and high-salt condiments (11, 12). NL accounted for 44% of the variance in eating behaviors (13). High nutritional literacy establishes the basis for individuals to adopt scientific stress-control behaviors by reinforcing their health perceptions.

Exposure to foods that conflict with personal dietary goals, together with social pressures, situational temptations, and lapses in self-regulation, can substantially elevate the likelihood of unhealthy eating in the immediate period that follows (14). The translation of dietary intentions into concrete actions is further shaped by contextual factors such as social cues, stress, and cravings, which may undermine adherence to recommended behaviors. Evidence indicates that nutritional knowledge and individual intentions serve as pivotal yet modifiable mediators in salt consumption (15). In line with the theory of planned behavior, intentions themselves are determined by personal attitudes and perceived social expectations, thereby exerting a direct influence on actual dietary practices (16). Intervention studies suggest that targeted educational programs can improve awareness and salt-related eating behaviors among individuals with hypertension; however, outcomes remain inconsistent across contexts. Importantly, knowledge of the health consequences associated with excessive dietary salt intake does not invariably result in reduced consumption. Khoram et al. (17) reported no association between knowledge regarding salt intake and actual reductions in use. Similarly, in a national survey, less than half of respondents who were familiar with salt-reduction initiatives reported actively applying them in practice (18). Together, these findings underscore the need to identify the principal determinants of salt-reduction behavior in order to design effective, evidence-based interventions for blood pressure control.

Bengbu, located along the Qinling–Huaihe Line—the conventional boundary between northern and southern China—presents epidemiological characteristics that make it a representative model of the nation's demographic transition (19). The region exhibits a distinctive confluence of northern and southern dietary and cultural practices. This study, conducted in Bengbu, aimed to investigate the association between NL and hypertension control and the potential chain mediating association of diet-control intention and salt-restriction tools utilization, providing theoretical support and empirical evidence for the development of nutritional intervention strategies and public health policies for hypertension.

## 2 Materials and methods

### 2.1 Participants

This cross-sectional survey was conducted in May 2023 in Bengbu, Anhui Province, China. A multistage cluster sampling design was adopted to select the study population. In consideration of the extensively documented disparities between urban and rural regions in China, urban and rural stratification was used. In the first stage, two urban districts and two rural townships were randomly identified as primary sampling units. In the second stage, two streets and two towns or villages were randomly selected from each selected unit, respectively. In the final stage, 110 households were randomly sampled within the chosen communities, and all eligible members of these households were invited to participate. Eligibility criteria were: age 18 years or older, conscious and able to communicate verbally without difficulty, and able to complete the questionnaire independently or with assistance from trained investigators. Participation was voluntary, and written informed consent was obtained from every respondent. Data collection was carried out using a structured questionnaire administered via face-to-face interviews, which included information on sociodemographic, lifestyle behaviors, and nutrition- and hypertension-related factors. The study was conducted in accordance with the Declaration of Helsinki and received ethical approval from the Ethics Committee of Bengbu Medical College (Approval No. 2021-099).

In this study, 904 individuals who had hypertension completed the survey. Thirty-eight (4.2%) were excluded because of incomplete NL and hypertension control effects. Consequently, the remaining 866 individuals were analyzed in this study. The characteristics of the missing samples were the same as those of the final samples.

### 2.2 NL assessment

The NL of participants was evaluated via the 12-item short-form NL scale (20). The instrument comprises two domains, i.e., nutrition cognition and nutrition skills, and six dimensions, i.e., knowledge, understanding, obtaining skills, applying skills, interactive skills and critical skills. Responses are provided on a five-point Likert scale, and the total score is calculated by summing the score of all items, with greater values reflecting better NL. Previous studies have demonstrated satisfactory validity of this instrument among Chinese adults (10, 21). The Cronbach's coefficient of the scale in this study was 0.861, with respective values of 0.823 for the nutrition cognition domain and 0.720 for the nutrition skills domains.

### 2.3 Diet-control intention and salt-restriction tool utilization

The participants' diet-control intentions were assessed based on their self-reported intention to adopt dietary behaviors that facilitate blood pressure control by means of the following question: “How about your intention to adopt dietary measures to control blood pressure?” The responses were then categorized into three classes: no intention, weak intention, and strong intention. Prior evidence indicates that clearly defined and unidimensional constructs such as behavioral intention can be validly captured by single-item instruments, with comparable predictive validity to multi-item scales (22). Given the simplicity of the construct and the need to minimize respondent burden, this study used this single item to measure diet-control intention. Salt-restriction tools utilization was measured by asking participants whether they used salt reduction tools (e.g., salt restriction spoons or rationing spoons). Response includes yes and no.

### 2.4 Self-rating effectiveness of hypertension control

Effectiveness of hypertension control was evaluated based on the individual's self-rating usual status of blood pressure monitoring and any comorbid conditions, including five categories, i.e., very good (normal blood pressure without complications), good (blood pressure is within the normal range at most of the time without complications), neutral (blood pressure is within the normal range at most of the time with complications), poor (blood pressure is high at most of the time without complications), and very poor (blood pressure is high at most of the time with complications).

### 2.5 Other variables

Potential confounders such as socio-demographic characteristics, lifestyle variables, and chronic disease status were adjusted to ensure result validity. Data collected included age (18–44, 45–64, or ≥65 years), sex (male or female), hukou category (rural or urban), marital status (married or other), educational attainment (primary school and below, junior high school, or senior high school and above), living status (living alone or non-living alone), type of occupational (farmer, leaver/retiree, or other), monthly income (< 1,000, 1,000–3,000, or >3,000 yuan), body mass index (BMI < 18.5, 18.5–23.9, 24.0–27.9, or >28.0 kg/m^2^), disease duration (0–5, 6–15, or >15 years), smoking status (never smoked, ever smoked, and currently smoked), drinking status (never smoked, ever smoked, and currently smoked), and exercise status (never exercised, ever exercised, and currently exercised), health self-assessment (excellent, good, fair, poor or very poor), and diabetes (yes or no).

### 2.6 Statistical analyses

Means with standard deviations (SD) was used to describe continuous variables, whereas frequencies and percentages were used to describe categorical variables. The normality of continuous variables was evaluated and independent-samples *t-*tests were employed to compare statistical differences between participants who did and did not use salt-restriction tools. The χ^2^ test was applied to test the association between categorical variables. NL was specified as a continuous predictor (range 12–60). Self-rating effectiveness of hypertension control (five-level Likert) was treated as an ordinal categorical variable.

To explore the relationships among NL, diet-control intention, salt-restriction tool utilization, and hypertension control effectiveness, binary logistic regression was applied to calculate odds ratios (OR) and corresponding 95% confidence intervals (CI) for the relationship between NL and salt-restriction tool utilization, and multinomial logistic regression was used to assess the associations of NL with diet-control intention and hypertension control effectiveness. Descriptive statistics and basic logistic regression analyses were implemented using the SPSS Statistics program (IBM). Two-sided *P*-values less than 0.05 were considered statistically significant.

Mediation models can be appropriately applied to cross-sectional data when the hypothesized relationships possess theoretical grounding and temporal plausibility (23, 24). Chained mediation analysis was conducted using a generalized structural equation modeling-based path model accommodating mixed variable types in Stata (Stata Corp, College Station, TX, USA). Chained mediation analysis was used with the Preacher and Hayes six-path framework to examine whether diet-control intention and salt-restriction tool utilization sequentially mediated the association between NL and hypertension control. Sex, age, hukou, marital status, education, living arrangement, occupation, monthly income, BMI, disease duration, smoking, drinking, exercise, self-rated health, and diabetes were included as exogenous predictors of all endogenous variables (M1, M2, and Y), ensuring mutual adjustment across paths. Mediation associations were estimated using bias-corrected 95% confidence intervals derived from 5,000 bootstrap samples, with significance indicated when the interval excluded zero. The model was saturated (df = 0), as all exogenous variables were allowed to covary freely without cross-equation constraints. Therefore, global fit indices (χ^2^, CFI, TLI, RMSEA) were not informative. Instead, model adequacy was evaluated using residual-based indices, yielding SRMR < 0.001 and coefficient of determination = 0.27. No evidence of multicollinearity (VIF < 5) or violation of normality assumptions was observed.

## 3 Results

### 3.1 General characteristics

Table 1 present participants' demographic characteristics. Of 866 individuals with hypertension, 426 demonstrated a strong intention to adopt a diet to control hypertension. This study found that hukou category and exercise are significantly correlated with the diet-control intention (*P* < 0.05). Additionally, 739 respondents reported not using any salt restriction tools. There were statistically significant differences in the salt-restriction tools utilization across hukou category, education, type of occupation, monthly income, exercise and health self-assessment (*P* < 0.001). Based on their self-rating effectiveness of hypertension control, 165 participants rated their blood pressure control as “excellent” and 391 as “good.” The results showed that hukou category, education, type of occupation, monthly income, exercise and health self-assessment were all significantly associated with self-rating effectiveness of hypertension control (*P* < 0.05). These results are presented in Table 1.

**Table 1:** Participants' diet-control intention, salt-restriction tools utilization and self-rating effectiveness of hypertension control.

**Baseline characteristic**	***N* (%)**	**Diet-control intention** ***N*** **(%)**			***X^2^***	**Salt-restriction tools**		***X^2^***	**Self-rating effectiveness of hypertension**					***X^2^***
						**utilization** ***N*** **(%)**			**control** ***N*** **(%)**					
		**Non-intentional**	**Weak intention**	**Strong intention**		**Yes**	**No**		**Excellent**	**Good**	**Fair**	**Poor**	**Very poor**	
Total	866 (100)	88 (10.2)	352 (40.6)	426 (49.2)		127 (14.7)	739 (85.3)		165 (19.1)	391 (45.2)	180 (20.8)	99 (11.4)	31 (3.6)	
**Sex**														
Male	367 (42.4)	41 (11.2)	151 (41.1)	175 (47.7)	0.973	51 (13.9)	316 (86.1)	0.301	72 (19.6)	159 (43.3)	87 (23.7)	40 (10.9)	9 (2.5)	5.61
Female	519 (57.6)	47 (9.4)	201 (40.3)	251 (50.3)		76 (15.2)	423 (84.8)		93 (18.6)	232 (46.5)	93 (18.6)	59 (11.8)	22 (4.4)	
**Age**														
18–44	21 (2.4)	4 (19.0)	5 (23.8)	12 (57.1)	5.38	5 (23.8)	16 (76.1)	2.295	4 (19.1)	10 (47.6)	3 (14.2)	4 (19.1)	0	12.216
45–64	238 (27.6)	26 (10.9)	86 (36.1)	126 (52.9)		39 (16.3)	199 (83.6)		54 (22.6)	95 (39.9)	43 (18.1)	35 (14.7)	11 (4.6)	
65–95	606 (70.0)	59 (9.7)	253 (41.7)	294 (48.5)		86 (14.2)	520 (85.8)		112 (18.4)	287 (47.4)	130 (21.5)	55 (9.1)	22 (3.6)	
**Hukou category**														
Urban	380 (43.9)	45 (11.8)	168 (44.2)	167 (43.9)	7.783^*^	76 (20.0)	304 (80.0)	15.399^***^	90 (23.7)	181 (47.6)	75 (19.7)	29 (7.6)	5 (1.3)	27.152^***^
Rural	486 (56.1)	43 (8.8)	184 (37.9)	259 (53.3)		51 (10.5)	435 (89.5)		75 (15.4)	210 (43.2)	105 (21.6)	70 (14.4)	26 (5.3)	
**Marital status**														
Married	856 (98.8)	87 (10.2)	346 (40.4)	423 (49.4)	1.69	126 (14.7)	730 (85.3)	0.176	163 (19.0)	389 (45.4)	176 (20.6)	98 (11.4)	30 (3.5)	4.402
Others	10 (1.2)	1 (10.0)	6 (60.0)	3 (30.0)		1 (10.0)	9 (90.0)		2 (20.0)	2 (20.0)	4 (40.0)	1 (10.0)	1 (10.0)	
**Education**														
Primary school and below	506 (58.4)	54 (10.7)	199 (39.3)	253 (50.0)	6.518	55 (10.9)	451 (89.1)	14.241^***^	73 (14.4)	233 (46.0)	112 (22.1)	65 (12.8)	23 (4.5)	27.103^***^
Junior High School	193 (22.3)	22 (11.4)	89 (46.1)	82 (42.5)		37 (19.2)	156 (80.8)		45 (23.3)	81 (42.0)	41 (21.2)	18 (9.3)	8 (4.1)	
High school and above	167 (19.3)	12 (7.2)	66 (38.3)	91 (54.5)		35 (21.0)	132 (79.0)		47 (28.1)	77 (46.1)	27 (16.2)	16 (9.8)	0	
**Living status**														
Living alone	124 (14.3)	11 (8.9)	59 (47.6)	54 (43.5)	2.886	17 (13.7)	107 (86.3)	0.106	23 (18.5)	53 (42.7)	29 (23.4)	14 (11.3)	5 (4.0)	0.762
Not living alone	742 (85.7)	74 (10.4)	293 (39.5)	372 (50.1)		110 (14.8)	632 (85.2)		142 (19.1)	338 (45.6)	151 (20.4)	85 (11.5)	26 (3.5)	
**Type of occupation**														
Farmers	310 (35.8)	26 (8.4)	124 (40.0)	160 (51.6)	6.244	32 (10.3)	278 (89.7)	16.859^***^	52 (16.8)	130 (41.9)	62 (20.0)	47 (15.2)	19 (6.1)	31.485^***^
Separated/retired staff	342 (39.5)	40 (11.7)	150 (43.9)	152 (44.4)		71 (20.8)	271 (79.2)		78 (22.8)	170 (49.7)	65 (19.0)	27 (7.9)	2 (0.6)	
Others	214 (24.7)	22 (10.3)	78 (36.4)	114 (53.3)		24 (11.2)	190 (88.8)		35 (16.4)	91 (42.5)	53 (24.8)	25 (11.7)	10 (4.7)	
**Monthly income**														
< 1,000	440 (50.8)	43 (9.9)	168 (38.2)	230 (51.9)	4.085	44 (10.0)	396 (90.0)	15.903^***^	59 (13.4)	203 (46.1)	101 (23.0)	52 (11.8)	25 (5.7)	33.686^***^
1,000–3,000	225 (25.9)	26 (11.7)	98 (43.7)	101 (44.6)		42 (18.7)	183 (81.3)		52 (23.1)	102 (45.3)	38 (16.9)	30 (13.3)	3 (1.3)	
>3,000	201 (23.2)	19 (9.5)	88 (43.7)	94 (46.8)		40 (19.9)	161 (80.0)		56 (27.9)	84 (41.8)	41 (20.4)	17 (8.5)	3 (1.5)	
**BMI**														
< 18.5	16 (1.8)	3 (18.8)	8 (50.0)	5 (31.3)	5.205	1 (6.3)	15 (93.8)	1.391	3 (18.8)	3 (18.8)	6 (37.5)	1 (6.3)	3 (18.8)	17.268
18.5–23.9	225 (26.0)	19 (8.4)	97 (43.1)	109 (48.4)		35 (15.6)	190 (84.4)		44 (19.6)	100 (44.4)	50 (22.2)	25 (11.1)	6 (2.7)	
24.0–28	388 (44.8)	45 (11.6)	150 (38.7)	193 (49.7)		54 (13.9)	334 (86.1)		71 (18.3)	179 (46.1)	76 (19.6)	47 (12.1)	15 (3.9)	
>28.0	237 (27.4)	21 (8.9)	97 (40.9)	119 (50.2)		37 (15.6)	200 (84.4)		47 (19.8)	106 (46.0)	48 (20.3)	26 (11.0)	7 (3.0)	
**Disease duration**														
0–5	296 (34.2)	29 (9.8)	126 (42.6)	141 (47.6)	0.775	49 (16.6)	247 (83.4)	2.885	58 (19.6)	132 (44.6)	60 (20.3)	38 (12.8)	8 (2.7)	7.958
6–15	338 (39.0)	34 (10.1)	135 (39.9)	169 (50.0)		41 (12.1)	297 (87.9)		67 (19.8)	158 (46.7)	63 (18.6)	33 (9.8)	17 (5.0)	
>15	232 (26.8)	25 (10.8)	91 (39.2)	116 (50.0)		37 (15.9)	195 (84.1)		40 (17.2)	101 (43.5)	57 (24.6)	28 (12.1)	6 (2.6)	
**Smoking status**														
Never smoked	603 (69.6)	54 (9.0)	239 (39.6)	310 (51.4)	6.225	92 (15.3)	511 (84.7)	3.939	120 (19.9)	272 (45.1)	118 (19.6)	72 (11.9)	21 (3.5)	11.125
Used to smoke	104 (12.0)	14 (13.5)	41 (39.4)	49 (47.1)		19 (18.3)	85 (81.7)		16 (15.4)	53 (51.0)	21 (20.2)	7 (6.7)	7 (6.7)	
Currently smoking	159 (18.4)	20 (12.6)	72 (45.3)	67 (42.1)		16 (10.1)	143 (89.9)		29 (18.2)	66 (41.5)	41 (25.8)	20 (12.6)	3 (1.9)	
**Drinking status**														
Never drank	524 (60.5)	48 (9.2)	207 (39.5)	269 (51.3)	3.093	77 (14.7)	447 (85.3)	0.13	97 (18.5)	242 (46.2)	106 (20.2)	59 (11.1)	21 (4.0)	5.421
Used to drink	124 (14.3)	14 (11.3)	54 (43.5)	56 (42.5)		17 (13.7)	107 (86.3)		23 (18.5)	51 (41.1)	31 (25.0)	13 (10.5)	6 (4.8)	
Currently drinking	218 (25.2)	26 (11.9)	91 (41.7)	101 (46.3)		33 (15.1)	185 (84.9)		45 (20.6)	98 (45.0)	43 (19.7)	28 (12.8)	4 (1.8)	
**Exercise**														
Never exercised	264 (30.5)	29 (11.0)	130 (49.2)	105 (39.8)	14.027^**^	27 (10.2)	237 (89.8)	6.199^*^	41 (15.5)	107 (40.5)	63 (23.9)	43 (16.3)	10 (3.8)	18.571^*^
Past exercise	68 (7.9)	7 (10.3)	25 (36.8)	36 (52.9)		10 (14.7)	58 (85.3)		10 (14.7)	33 (48.5)	14 (20.6)	6 (8.8)	5 (7.4)	
Current exercise	534 (61.7)	52 (9.7)	197 (36.9)	285 (53.4)		90 (16.9)	444 (83.1)		114 (21.3)	251 (47.0)	103 (19.3)	50 (9.4)	16 (3.0)	
**Health self-assessment**														
Excellent	40 (4.6)	6 (15.0)	13 (32.5)	21 (52.5)	4.425	13 (32.5)	27 (67.5)	20.828^***^	21 (52.5)	12 (30.0)	3 (7.5)	2 (5.0)	2 (5.0)	82.704^***^
Good	114 (13.2)	11 (9.6)	41 (36.0)	62 (54.4)		24 (21.1)	90 (78.9)		33 (28.9)	47 (41.2)	22 (19.3)	10 (8.8)	2 (1.8)	
Fair	216 (24.9)	19 (8.8)	93 (43.1)	104 (48.1)		28 (13.0)	188 (87.0)		44 (20.4)	110 (50.9)	35 (16.2)	22 (10.2)	5 (2.3)	
Poor	346 (40.0)	37 (10.7)	146 (42.4)	163 (47.1)		51 (14.7)	295 (85.3)		53 (15.3)	169 (48.8)	76 (22.0)	40 (11.6)	8 (2.3)	
Very poor	150 (17.3)	15 (10.0)	59 (39.3)	76 (50.7)		11 (7.3)	139 (92.7)		14 (9.3)	53 (35.3)	44 (29.3)	25 (16.7)	14 (9.3)	
**Diabetes**														
Yes	181 (20.9)	19 (10.5)	67 (37.0)	95 (52.5)	1.272	29 (16.0)	152 (84.0)	0.337	32 (17.7)	73 (40.3)	44 (24.3)	22 (12.2)	10 (5.5)	5.294
No	685 (79.1)	69 (10.1)	285 (41.6)	331 (48.3)		98 (14.3)	587 (85.7)		133 (19.4)	318 (46.4)	136 (19.9)	77 (11.2)	21 (3.1)	

^*^P < 0.05, ^**^P < 0.01, ^***^P < 0.001.

### 3.2 Score of NL according to salt-restriction tools utilization

On average, hypertensive patients using salt restriction tools had higher NL scores than those not using such tools (*P* < 0.001). Similarly, statistically significant differences were observed between hypertensive patients using salt restriction tools and those not using them in terms of average NL scores for nutritional cognition, nutritional skill, knowledge, understanding, obtaining skills, applying skills, interactive skills, and critical skills (*P* < 0.001). Calculations of effect sizes revealed Cohen's *d* values ranged from 0.40 to 0.72, indicating a medium to substantial magnitude of difference between the groups. Detailed data are presented in Table 2.

**Table 2:** Score of NL according to salt-restriction tools utilization.

**Variables**	**Salt-restriction tools utilization (mean** ±**SD)**		***t***	***P*-value**	**Cohen's *d* (95 % CI)**
	**Yes**	**No**			
Nutrition literacy	40.57 ± 7.93	34.90 ± 7.88	7.452	< 0.001	−0.72 (−0.91, −0.53)
Nutrition cognition	14.91 ± 2.96	13.08 ± 3.09	6.389	< 0.001	−0.60 (−0.79, −0.41)
Nutrition skill	25.67 ± 5.72	21.83 ± 5.35	7.059	< 0.001	−0.71 (−0.90, −0.52)
Knowledge	8.40 ± 1.46	7.61 ± 1.52	5.633	< 0.001	−0.53 (−0.72, −0.34)
Understanding	6.50 ± 2.17	5.47 ± 2.15	4.983	< 0.001	−0.48 (−0.67, −0.29)
Obtaining skills	5.86 ± 2.34	4.92 ± 2.05	4.668	< 0.001	−0.45 (−0.64, −0.26)
Applying skills	5.70 ± 2.04	4.66 ± 1.75	5.421	< 0.001	−0.58 (−0.77, −0.39)
Interactive skills	7.20 ± 1.73	6.50 ± 1.77	4.199	< 0.001	−0.40 (−0.59, −0.21)
Critical skills	6.91 ± 1.74	5.76 ± 1.77	6.871	< 0.001	−0.65 (−0.84, −0.46)

### 3.3 Relationship of NL with the diet-control intention, salt-restriction tools utilization and self-rating effectiveness of hypertension control

After adjusting for potential confounders, NL was linked to the diet-control intention (OR = 1.11, 95% CI: 1.09, 1.14), the salt-restriction tools utilization (OR = 1.08, 95% CI: 1.05, 1.12), and self-rating effectiveness of hypertension control (OR = 1.04, 95% CI: 1.02, 1.06). We found significant positive associations between NL and diet-control intention, as well as the salt-restriction tools utilization, in both domains and across the six NL dimensions. Positive correlations were also found between self-rating effectiveness of hypertension control outcomes and both domains, as well as across the four NL dimensions. However, no correlation was found for “Obtaining skills” (OR = 1.06, 95% CI: 0.98, 1.15). and “Applying skills” (OR = 1.06, 95% CI: 0.98, 1.15). dimensions. The detailed results are shown in Table 3.

**Table 3:** Association of nutritional literacy and diet-control intention, salt-restriction tools utilization, and self-rating effectiveness of hypertension control.

**Variables**	**Diet-control intention OR (95% CI)**	**Salt-restriction tools utilization OR (95% CI)**	**Self-rating effectiveness of hypertension control OR (95% CI)**
Nutrition literacy	1.112 (1.088–1.137)^***^	1.086 (1.053–1.119)^***^	1.035 (1.015–1.055)^***^
Nutrition cognition	1.290 (1.222–1.361)^***^	1.181 (1.096–1.271)^***^	1.085 (1.035–1.137)^***^
Nutrition skill	1.144 (1.109–1.180)^***^	1.123 (1.075–1.174)^***^	1.047 (1.018–1.076)^**^
Knowledge	1.662 (1.502–1.840)^***^	1.443 (1.238–1.682)^***^	1.178 (1.076–1.289)^***^
Understanding	1.261 (1.171–1.359)^***^	1.167 (1.050–1.297)^**^	1.087 (1.015–1.163)^*^
Obtaining skills	1.258 (1.162–1.362)^***^	1.163 (1.045–1.294)^**^	1.065 (0.992–1.144)
Applying skills	1.227 (1.126–1.337)^***^	1.267 (1.126–1.427)^***^	1.062 (0.982–1.148)
Interactive skills	1.299 (1.193–1.414)^***^	1.212 (1.072–1.371)^**^	1.089 (1.008–1.178)^*^
Critical skills	1.404 (1.290–1.528)^***^	1.353 (1.197–1.529)^***^	1.158 (1.072–1.252)^***^

^*^P < 0.05, ^**^P < 0.01, ^***^P < 0.001. OR, odds ratio; 95% CI, 95% confidence interval. Multiple logistic regression was used to calculate the diet-control intention OR when adjusting for sex, age, hukou category, marital status, education, living status, type of occupation, monthly income, body mass index, disease duration, smoking status, drinking status, exercise, health self-assessment, and diabetes. Binary logistic regression was used to calculate the salt-restriction tools utilization OR when adjusting for sex, age, hukou category, marital status, education, living status, type of occupation, monthly income, body mass index, disease duration, smoking status, drinking status, exercise, health self-assessment, and diabetes. Multiple logistic regression was used to calculate the self-rating effectiveness of hypertension control OR when adjusting for sex, age, hukou category, marital status, education, living status, type of occupation, monthly income, body mass index, disease duration, smoking status, drinking status, exercise, health self-assessment, and diabetes.

### 3.4 Mediation analysis

**Figure 1:**
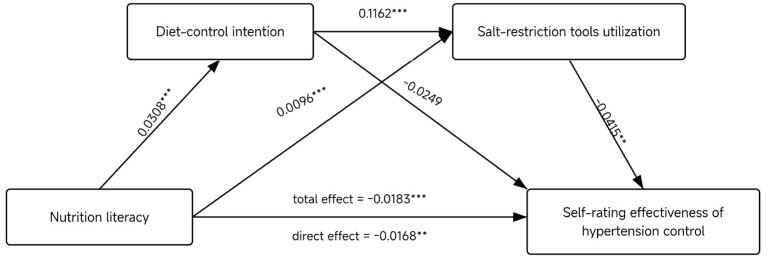
Mediation model of diet-control intention and salt-restriction tools utilization between nutrition literacy and self-rating effectiveness of hypertension control. ^**^P < 0.01, ^***^P < 0.001.

The results of the mediation analysis are presented in Table 4 and Figure 1. Since higher scores on the outcome variable denote poorer control, the observed negative coefficients suggest an improvement in hypertension management. The analysis demonstrated a significant total association of NL with self-rating effectiveness of hypertension control (β = −0.0183, 95% CI: −0.0286, −0.0081) and a significant direct association (β = −0.0168, 95% CI: −0.0276, −0.0059) after adjusting for mediators, suggesting an independent protective role of NL. Bootstrapping confirmed two significant indirect pathways: one through salt-restriction tools utilization (β = −0.0026, 95% CI: −0.0046, −0.0006), and a serial mediation pathway wherein NL increased diet-control intention, which in turn enhanced salt-restriction tools utilization, and ultimately leading to better hypertension control (β = −0.0008, 95% CI: −0.0015, −0.0001).

**Table 4:** Diet-control intention and salt-restriction tools utilization mediating models between nutrition literacy and self-rating effectiveness of hypertension control.

**Pathway**	**β**	**SE**	**BootLLCl**	**BootULCl**
Total association	−0.0183	0.0052	−0.0286	−0.0081
Direct association	−0.0168	0.0055	−0.0276	−0.0059
NL → diet-control intention	0.0308	0.0031	0.0247	0.037
NL → salt-restriction tools utilization	0.0096	0.0017	0.0062	0.013
Diet-control intention → salt-restriction tools utilization	0.1162	0.0182	0.0804	0.1521
Diet-control intention → self-rating effectiveness of hypertension control	−0.0249	0.0237	−0.0714	0.0216
Salt-restriction tools utilization → self-rating effectiveness of hypertension control	−0.0415	0.0125	−0.066	−0.017
**Indirect association**				
Total indirect association	−0.0023	0.0019	−0.0061	0.0014
NL → diet-control intention → self-rating effectiveness of hypertension control	0.0011	0.0017	−0.0022	0.0044
NL → salt-restriction tools utilization → self-rating effectiveness of hypertension control	−0.0026	0.001	−0.0046	−0.0006
NL → diet-control intention → salt-restriction tools utilization → self-rating effectiveness of hypertension control	−0.0008	0.0003	−0.0015	−0.0001

BootLLCI, bootstrapping lower limit confidence interval; BootULCI, bootstrapping upper limit confidence interval; SE, standard error; Effect, standardized regression coefficient.

## 4 Discussion

This study was the first to investigate the sequential mediating roles of diet-control intention and salt-restriction tools utilization in the relationship between NL and effectiveness of hypertension control. The findings showed NL has a significant positive association with hypertension control, and this relationship was chain-mediated by diet-control intention and salt-restriction tools utilization. Improving patients' nutritional literacy is conductive to encouraging them to control their diet, and utilizing salt restriction tools to achieve the goal of controlling hypertension.

Among individuals with hypertension in our sample, merely 19.1% indicated that their blood pressure is well-controlled. The results align with international evidence showing that the proportion of individuals attaining adequate blood pressure control remains low, at about 23% among women and 18% among men with hypertension (25). Although the control rate of Chinese hypertensive patients has improved significantly, the overall rate remains low at just 16.8% (10). Unfortunately, the overall state of hypertension control was poor. Poor blood pressure control significantly increases the risk of stroke and renal failure, and is strongly associated with cognitive impairment and dementia (26, 27). Improving the management of blood pressure in hypertensive populations should be recognized as a pressing public health priority in China.

The findings of this study indicate that subjects with higher levels of NL exhibit superior blood pressure control. NL has been recognized as an important determinant of healthy dietary behaviors (28). The enhancement of nutritional literacy has been demonstrated to foster the development of beneficial dietary habits, thereby leading to a substantial enhancement in the quality of one's diet (29). Moreover, accumulating evidence suggests that following a nutrient-rich, well-balanced diet exerts a direct regulatory influence on blood pressure, making it a pivotal strategy for effective hypertension control (30). The role of NL in the management of hypertension is contingent on the combined associations of multiple fields and dimensions. NL, comprising factual knowledge and understanding, provides patients with the rationale for modifying their diet. In a cohort of 828 working-age adults in Ireland, participants with the greatest nutrition knowledge showed a 5.8-fold higher likelihood of adhering to the DASH diet and had mean systolic blood pressure values about 2 mmHg lower compared with those possessing the least knowledge (31). It is also imperative to emphasize the significance of nutritional skills. Interactive and critical skills have been shown to independently predict positive self-management behaviors in hypertensive patients, indirectly promoting blood pressure control. As previously stated, cognition provides the underlying rationale, while skills facilitate the execution of specific actions, collectively mediating the pathway from high nutrient deficiency to sustained blood pressure control.

Sodium reduction is broadly regarded as a highly feasible and cost-effective public health measure in the prevention and management of hypertension and its related cardiovascular conditions. Various countries have adopted distinct measures to promote population-level salt reduction. For example, the United States has reformulated major food categories with the aim of lowering overall sodium intake (32), while South Africa has implemented mandatory regulations that establish sodium reduction targets for foods (33). In China The “Salt Control Spoon Project,” initiated in 2008, distributed complimentary, calibrated salt spoons with the objective of assisting residents in moderating their salt intake during daily culinary activities. Within the Chinese population, randomized controlled trials have demonstrated that interventions such as the distribution of salt-measuring spoons in combination with health education interventions can lead to meaningful reductions in dietary salt consumption, accompanied by improvements in blood pressure control (34). Despite the recognized benefits of sodium reduction, findings from our study revealed that merely 14.7% of hypertensive participants made use of salt-restriction tools. The extant evidence suggests that such tools, namely simple, low-cost behavioral interventions, have the capacity to assist individuals in intuitively regulating their salt intake, thereby fostering a potential decrease in blood pressure levels (35). Therefore, strengthening the availability and social acceptance of salt-restriction tools should be considered as a critical public health strategy for advancing hypertension prevention and control in the broader population. It is important to note that the combination of salt-limiting tools with comprehensive measures, such as nutrition education and health promotion, often results in superior prevention and treatment outcomes (8, 35).

It is evident that dietary control constitutes a primary component in the management of hypertension. In a study of African American elderly patients with hypertension, although many patients were aware of the impact of diet on blood pressure, their actual intention to control their diet was not strong, which was related to various factors such as personal lifestyle habits and dietary preferences (36). In this study, 49.2% of participants expressed a strong willingness to adopt dietary behaviors to control hypertension. Patients who possess unwavering and resolute intentions to regulate their dietary intake are more inclined to proactively seek out and utilize tools designed to reduce salt intake in their daily lives (37). According to the theory of planned behavior, an individual's behavioral intention constitutes the fundamental impetus for behavioral change (16). Consistent with this evidence, a large-scale, government-led, population-based intervention conducted in Shandong Province, China, significantly reduced the intake of dietary sodium and achieved modest population-level reductions in blood pressure (38). This result indicates that dietary regulation intentions serve as a critical mediator connecting the utilization of salt-control devices with hypertension prevention and management outcomes, thereby establishing them as a pivotal factor in achieving a positive feedback loop in the management of hypertension through dietary means. Consequently, endeavors must be undertaken to fortify patients' dietary self-regulation intentions to enhance comprehensive hypertension prevention and management strategies.

The present study indicates that NL exerts a significant association on individuals' intentions to regulate their dietary behavior and their utilization of salt-restriction tools. Intervention strategies aimed at enhancing behavioral intentions, such as the establishment of goals, the improvement of self-efficacy, and the creation of a supportive environment, may facilitate the translation of nutritional knowledge into healthy dietary behaviors (39). Patients with high NL are more likely to have strong dietary control intentions, which in turn motivate them to actively use salt-restriction tools, manage salt intake scientifically, optimize dietary structure, and ultimately achieve good blood pressure control outcomes. Following modifications to their dietary habits, hypertensive patients exhibited a significant reduction in blood pressure levels (30). The incorporation of NL enhancement into public health intervention strategies has the potential to provide robust support for the prevention and control of hypertension and other chronic diseases, thereby promoting the sustainable development of public health initiatives.

A single cross-sectional blood pressure measurement does not adequately capture the daily blood pressure variations in individuals with hypertension, moreover, there is a large fluctuation in their 24-h daily blood pressure. Therefore, although the participants' blood pressure values were measured in this study, their self-reported usual state of blood pressure monitoring and comorbidities were used as the outcome variable. This may more truly reflect the long-term blood pressure control status of hypertensive patients and their perceived treatment effectiveness.

Several limitations should be acknowledged. Owing to its cross-sectional nature, this study cannot determine causality; prospective cohort or longitudinal intervention studies are warranted to further clarify the sustained impact of nutritional literacy on hypertension management. Moreover, as the sample was confined to Bengbu, Anhui Province, the generalizability of the results is limited. Future investigations incorporating broader and more diverse populations will strengthen the external validity of these findings.

## 5 Conclusion

NL significantly linked with blood pressure control in hypertensive population, and this association operates both directly and indirectly through a chain mediating pathway of diet-control intention and salt-restriction tool utilization. Nevertheless, the uptake of salt-restriction tools in this population was low, highlighting a gap between awareness and practice. Integrating nutritional literacy enhancement with strategies that strengthen dietary self-regulation and promote wider adoption of salt-restriction tools may provide more effective and sustainable approaches for hypertension management.

## Data Availability

The original contributions presented in the study are included in the article/supplementary material, further inquiries can be directed to the corresponding author.
